# Identification of a DNA Damage Response-Related Prognostic Signature and Functional Validation of SMC3 in Glioma

**DOI:** 10.3390/ijms27219958

**Published:** 2026-11-06

**Authors:** Jiqiang Ma, Chunyu Zhang, Jing Zhang, Chunlong Zhong

**Affiliations:** 1 Department of Neurosurgery, Shanghai East Hospital, Nanjing Medical University, Nanjing 211166, China; 2 Department of Neurosurgery, Shanghai East Hospital, School of Medicine, Tongji University, Shanghai 200092, China; 3 State Key Laboratory of Cardiovascular Diseases and Medical Innovation Center, Shanghai East Hospital, School of Medicine, Tongji University, Shanghai 200092, China

**Keywords:** glioma, DDR, prognosis, immune microenvironment, SMC3

## Abstract

DNA damage response (DDR) deficiencies are linked to tumorigenesis and progression. However, the association of DDR with prognosis and therapeutic response in glioma has not been fully illustrated. TCGA and CGGA databases were used to acquire RNA-seq and clinical data about gliomas. LASSO-Cox regression was introduced to establish a DDR-based risk signature. The single-sample gene set enrichment analysis (ssGSEA) algorithm was used to estimate immune infiltrates. The performance of the signature and nomogram in predicting survival was evaluated using ROC curves. Connectivity Map (CMap) analysis was used to nominate candidate compounds, and pRRophetic was used to predict drug sensitivity. Through LASSO-Cox regression, we developed a prognostic risk score based on five DDR-related genes (GADD45G, HDAC1, RRM2, SMC3, and WEE1). Higher risk scores were associated with shorter overall survival and remained significant in multivariable Cox analyses. A prognostic nomogram achieved 1-, 3-, and 5-year AUCs of 0.901, 0.936, and 0.903 in TCGA and 0.776, 0.816, and 0.802 in CGGA, respectively. High-risk tumors showed enrichment of TNFα signaling via NFκB and IL6/JAK/STAT3 gene sets, together with differences in inferred immune cell-associated features and immune checkpoint gene expression. Computational drug sensitivity analyses associated higher risk scores with lower predicted IC_50_ values for GSK269962A, pazopanib, rapamycin, and NVP-BEZ235, providing hypotheses for subsequent experimental validation. SMC3 overexpression inhibited glioma cell proliferation and migration in vitro. The DDR-related signature showed prognostic value and associations with immune-related transcriptional features in glioma, with in vitro evidence suggesting a functional role for SMC3.

## 1. Introduction

Representing roughly 80% of all central nervous system (CNS) malignancies, gliomas remain characterized by high mortality and adverse prognoses [1,2,3]. Gliomas encompass biologically and clinically distinct tumor entities with markedly different outcomes. Adult-type gliomas comprise three principal entities under WHO CNS5: astrocytoma, IDH-mutant; oligodendroglioma, IDH-mutant and 1p/19q-codeleted; and glioblastoma, IDH-wildtype [4]. These entities differ in their molecular characteristics, clinical course, and prognosis. IDH-mutant astrocytomas may progress to higher grades but remain diagnostically distinct from IDH-wildtype glioblastoma [5]. Prognostic signatures should therefore be evaluated within the framework of integrated molecular classification [6]. Consequently, identifying novel and robust biomarkers remains essential for optimizing patient risk stratification and enhancing prognostic precision.

Various endogenous and exogenous stressors continuously challenge DNA integrity, necessitating an efficient DNA damage response (DDR) to preserve genomic stability [7]. Impaired DDR mechanisms facilitate the accumulation of mutations, accelerating tumor evolution. For instance, defective nucleotide excision repair in xeroderma pigmentosum leads to ultraviolet (UV)-induced skin cancer, with UV exposure causing extensive point mutations and early tumor formation [8,9]. Tumors with DDR deficiencies often exhibit heightened sensitivity to genotoxic treatments like chemotherapy and radiotherapy, offering potential as predictive markers for personalized therapies [10,11,12]. Alkylating chemotherapy via temozolomide (TMZ) remains the frontline treatment for glioma, yet its efficacy is severely compromised by MGMT-driven DNA repair [13]. The DDR-mediated chemoresistance inevitably leads to dismal overall survival, underscoring the imperative to identify alternative candidate targets and precise prognostic biomarkers.

DDR and immune responses appear to be related and might have synergistic effects, and insights into the correlation of both might increase the clinical efficacy of immunotherapy [14]. Evidence indicates that therapies aimed at targeting DDR can bolster anti-tumor immune responses through various mechanisms by elevating mutagenicity and promoting genomic instability [15]. In addition, combined inhibition of DNA repair factors, such as USP1 and PARP, can activate STING signalling and promote immunogenic cell death, thereby enhancing antitumour immunity [16]. In a BRCA1-deficient mouse model of ovarian cancer, combined CTLA-4 blockade and PARP inhibition exerted synergistic antitumor effects, promoting immune-mediated tumour clearance and prolonging survival [17]. Meanwhile, the DDR can act as a therapeutic opportunity for human cancers [18] and predict prognosis, antitumor immune activity, and adjuvant response in non-small cell lung cancer (NSCLC) and colorectal cancer [19,20]. However, relevant research on glioma is lacking.

A prognosis model integrating transcriptome and clinical data enhances predictive accuracy and holds great clinical significance. The present research developed a DDR-based glioma prognostic risk score system. The gliomas had distinct clinical outcomes and tumour microenvironment (TME) features depending on the risk group. The evaluation of DDR-related scores might enhance clinical prognostic management and assist in directing personalised medical treatment.

## 2. Results

### 2.1. Differentially Expressed Genes (DEGs) Associated with DDR

**Figure 1:**
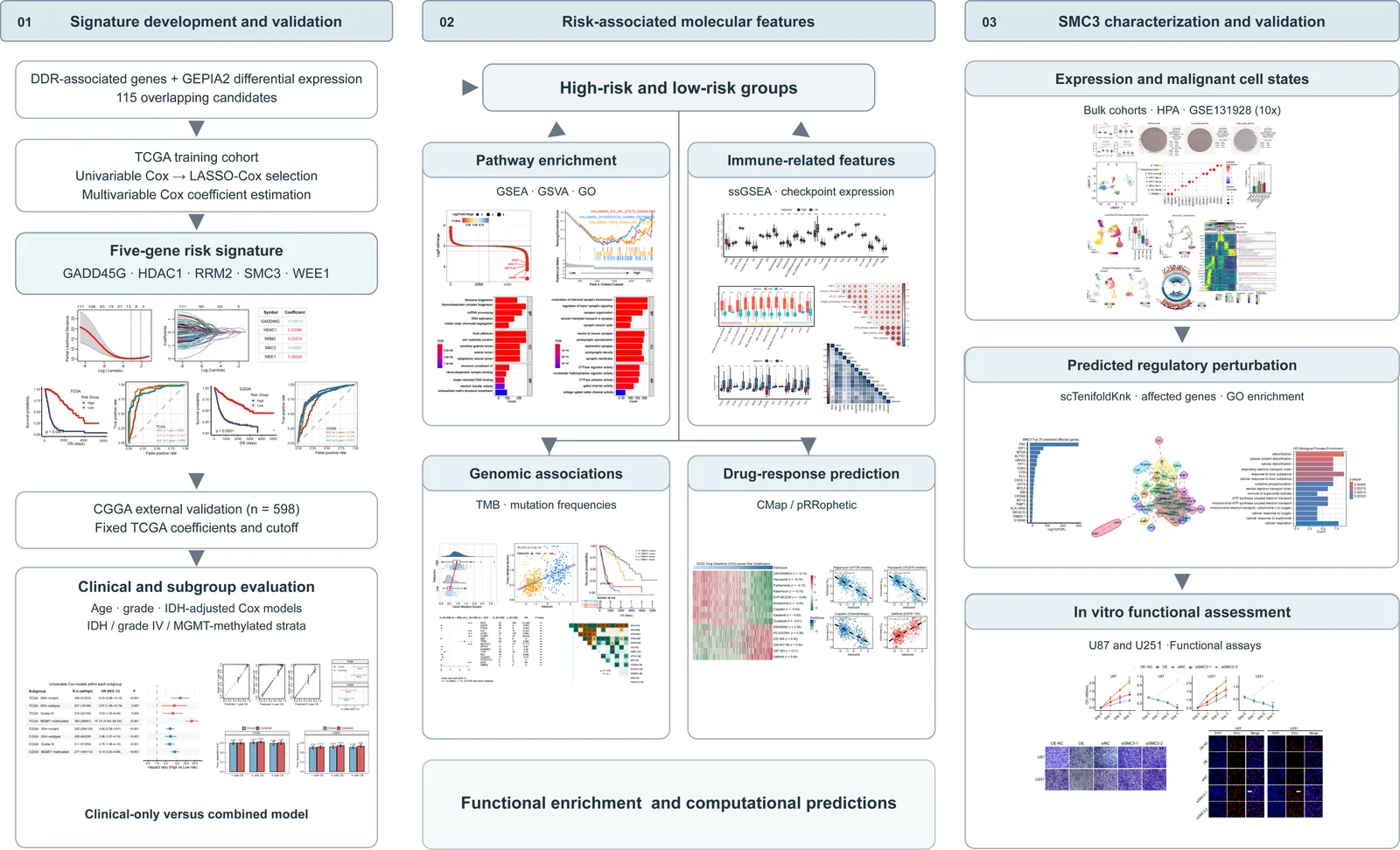
Overview of the research workflow.

**Figure 2:**
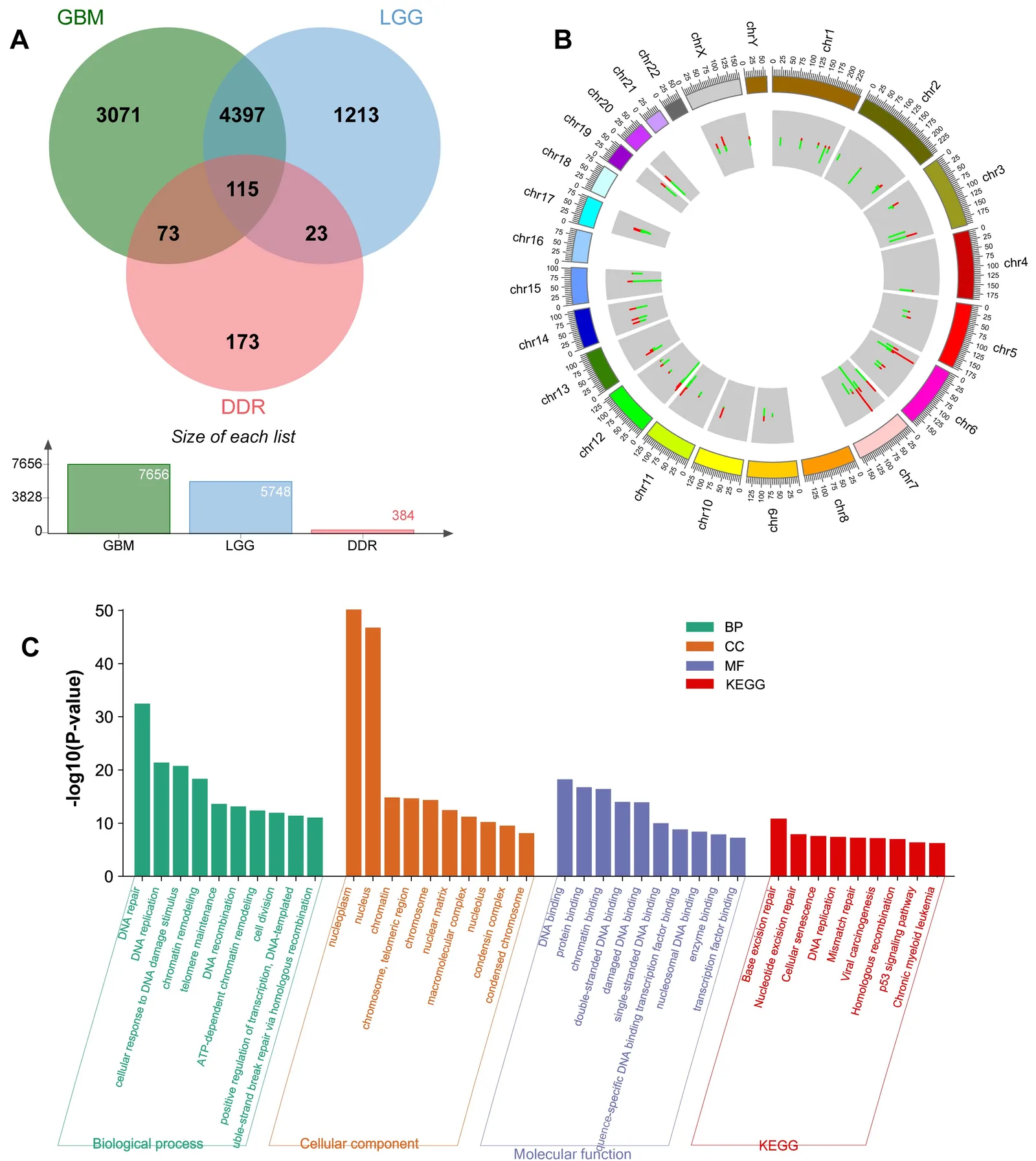
(**A**) Identification of differentially expressed genes (DEGs) related to DDR. (**B**) Circos plot of chromosome distribution of DEGs. The green bar represents the fold change in DEGs in LGG samples, and the red bar represents the fold change in DEGs in GBM samples. (**C**) Bar plot of enrichment analysis of DEGs.

The overall study workflow is illustrated in Figure 1. By interrogating the GEPIA2 database, a total of 115 cross-validated DNA damage response (DDR)-related molecules exhibiting differential expression between glioma specimens and non-tumor brain controls were identified, with their intersection harmonized via the ‘jvenn’ online interface [21]. Comparing the mRNA expression levels of 115 DDR-associated factors in nontumor brain samples, all 115 genes were found at higher expression levels in glioma tissues (Figure 2A and Table S1). To observe the genetic variation in DDR molecules in cancer, we visualized the mutant 113 genes and the corresponding chromosome positions (Figure 2B). Then, enrichment analysis of the 115 markers revealed that DDR-related processes were upregulated, such as DNA recombination, chromatin remodeling, damaged DNA binding, and DNA repair. Meanwhile, KEGG analysis demonstrated that mismatch repair and the p53 signaling pathway were relatively elevated (Figure 2C).

### 2.2. Development of a Risk Signature for Glioma

**Figure 3:**
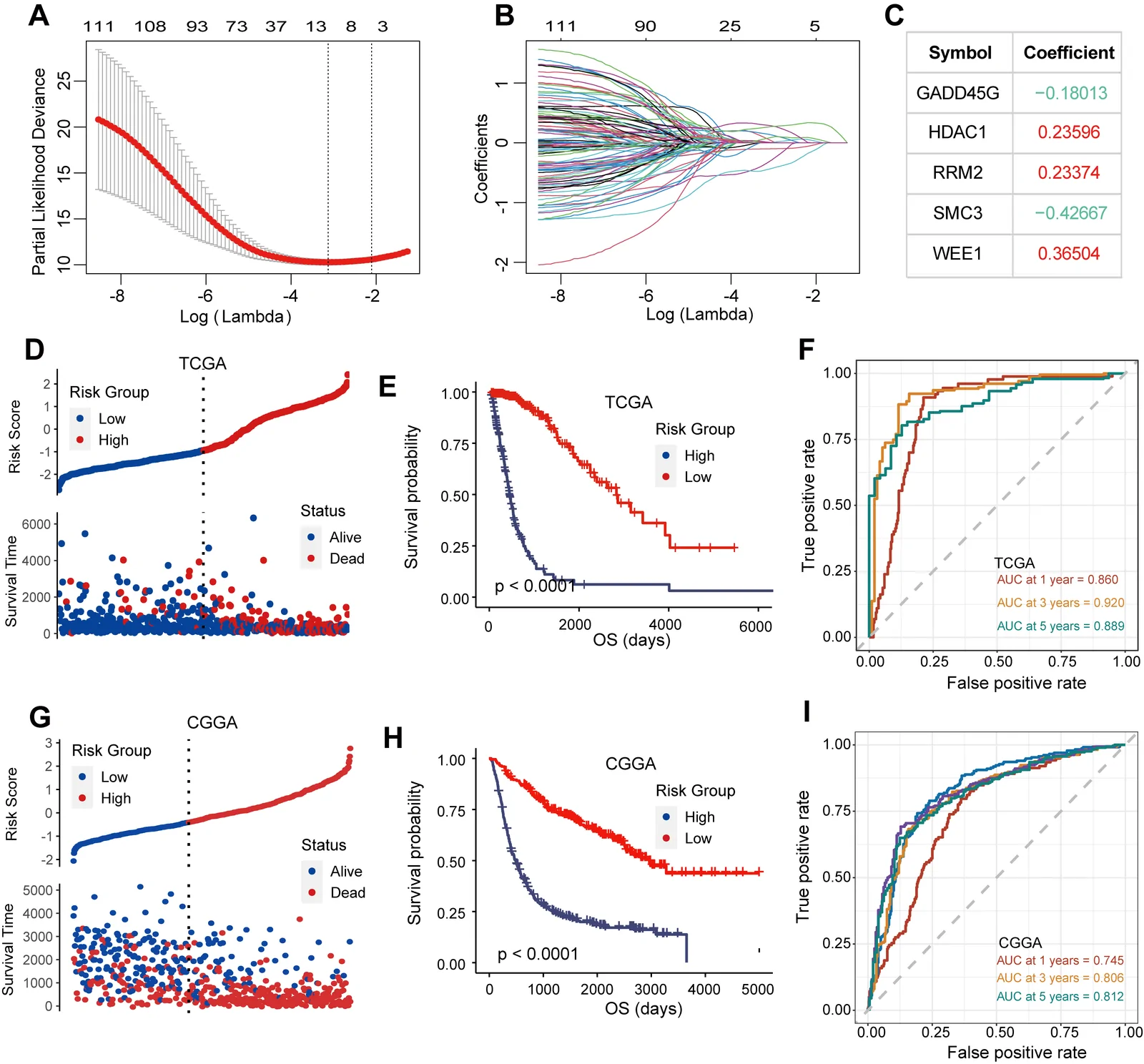
(**A**,**B**) LASSO Cox regression analysis of DEGs was used to shrink the variates. (**C**) The coefficients of 5 genes calculated by regression analysis. (**D**,**G**) The dotplot of the association of the risk score and survival status. (**E**,**H**) K-M plots demonstrated survival differences among gliomas after grouping by risk score. (**F**,**I**) ROC analysis of risk score in predicting glioma prognosis.

The 115 differentially expressed candidates underwent dimension reduction using the LASSO-Cox penalty method. Ultimately, 5 core predictive factors were filtered out, including GADD45G (Growth Arrest and DNA Damage Inducible Gamma), HDAC1 (Histone Deacetylase 1), RRM2 (Ribonucleotide Reductase Regulatory Subunit M2), SMC3 (Structural Maintenance of Chromosomes 3), and WEE1 (WEE1 G2 Checkpoint Kinase) (Figure 3A,B). The coefficients of the included genes were displayed in Figure 3C. Using the TCGA-trained model, the risk score was calculated as follows: Risk Score = (−0.18013 × Exp_GADD45G_) + (0.23596 × Exp_HDAC1_) + (0.23374 × Exp_RRM2_) + (−0.42667 × Exp_SMC3_) + (0.36504 × Exp_WEE1_) (Figure 3C). The strong co-expression relationship was found between SMC3 and other risk genes, particularly HDAC1 (r = 0.79), WEE1 (r = 0.70), and RRM2 (r = 0.61). To further evaluate the individual prognostic associations of SMC3 and GADD45G, both of which exhibited negative coefficients in the LASSO-Cox model, we performed additional K–M survival analyses and univariable Cox proportional hazards regression in the TCGA and CGGA cohorts. K–M analysis demonstrated that higher SMC3 expression was associated with significantly improved overall survival in both TCGA (log-rank *p* < 0.001) and CGGA (log-rank *p* = 0.013) (Figure S1A,B). Consistently, univariable Cox regression showed favourable prognostic associations for SMC3 in TCGA (HR = 0.376, 95% CI: 0.269–0.531) and CGGA (HR = 0.788, 95% CI: 0.652–0.952). Similar findings were observed for GADD45G, with higher expression associated with improved overall survival in both cohorts (both log-rank *p* < 0.001, Figure S1C,D). Univariable Cox regression further demonstrated favorable associations for GADD45G in TCGA (HR = 0.418, 95% CI: 0.289–0.604) and CGGA (HR = 0.665, 95% CI: 0.544–0.813) (Figure S1E). The favorable association aligns with a recent study demonstrating the tumor-suppressive role of GADD45G in glioma [22].

### 2.3. The DDR-Related Signature Performed a Prognostic Role in Glioma

**Figure 4:**
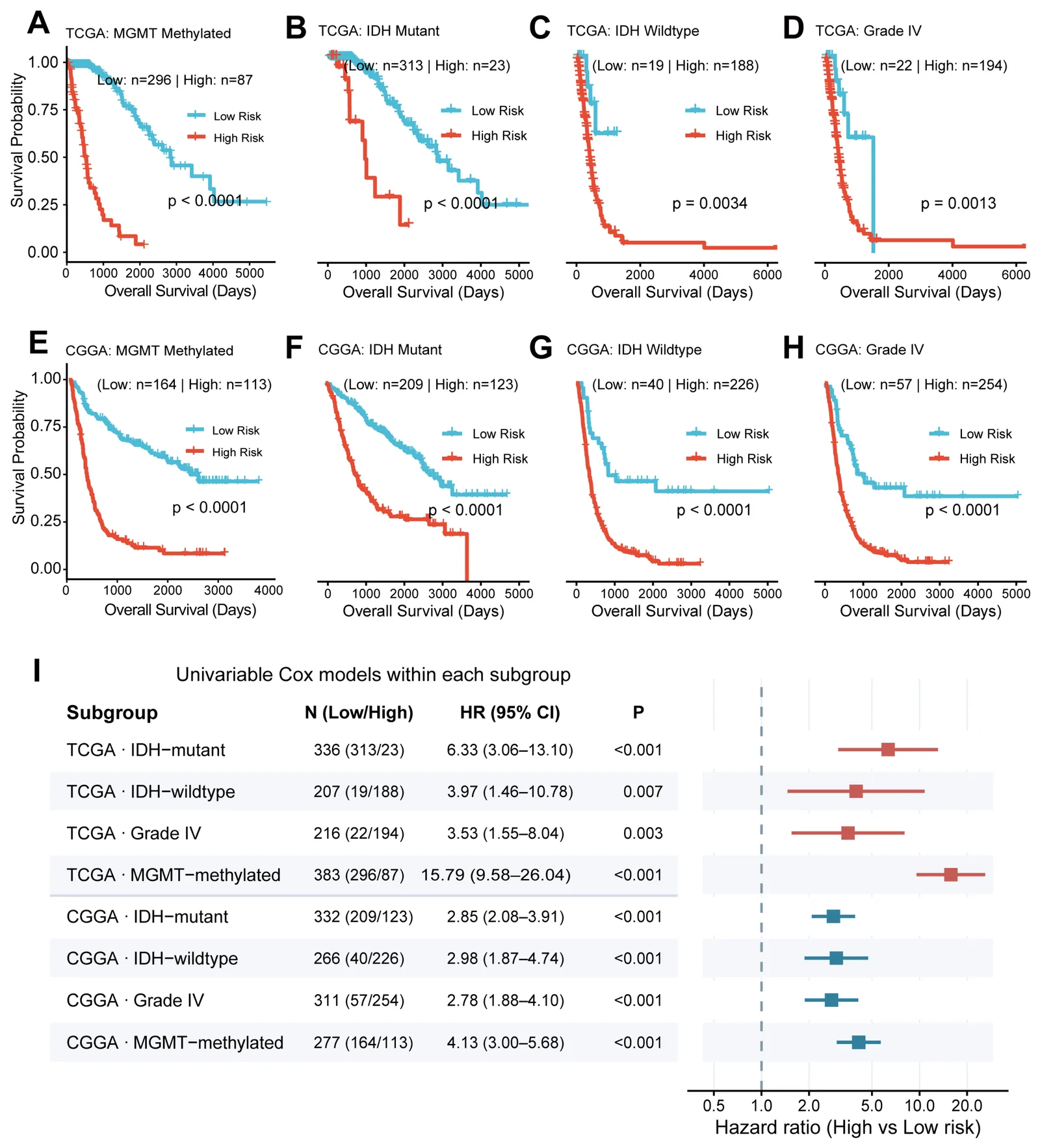
Subgroup survival analyses of the five-gene DDR-related risk signature in TCGA and CGGA. (**A**–**D**) Kaplan–Meier curves comparing overall survival between high- and low-risk patients within the MGMT promoter-methylated (**A**), IDH-mutant (**B**), IDH-wildtype (**C**), and grade IV (**D**) subgroups in TCGA. (**E**–**H**) Corresponding analyses in CGGA for the MGMT promoter-methylated (**E**), IDH-mutant (**F**), IDH-wildtype (**G**), and grade IV (**H**) subgroups. (**I**) Forest plot of subgroup-specific univariable Cox analyses comparing high- versus low-risk patients. Squares indicate hazard ratios (HRs), horizontal lines represent 95% confidence intervals (CIs), and the dashed vertical line denotes HR = 1. N indicates the number of patients, with low-/high-risk counts shown in parentheses.

We reclassified the cohorts according to the WHO CNS5 framework and IDH status. Stratified K–M analyses further confirmed significant overall survival differences across risk groups combined with age or sex (all log-rank *p* < 0.0001; Figure S2A–D). Risk scores correlated significantly with WHO grade, age, and IDH status, but were independent of sex (Table S2). To further evaluate the prognostic performance of the five-gene DDR-related signature within specific molecular and histological subgroups, separate Kaplan–Meier survival analyses were conducted. The five-gene signature retained its prognostic value across molecular and grade-defined subsets: high-risk status conferred inferior overall survival in MGMT-methylated and IDH-mutant groups (both log-rank *p* < 0.0001), as well as in IDH-wildtype (*p* = 0.0034) and grade IV (*p* = 0.0013) cohorts (Figure 4A–D). These findings were corroborated in the independent CGGA cohort (all log-rank *p* < 0.0001; Figure 4E–H). Cox modeling yielded hazard ratios of 3.53–15.79 (TCGA) and 2.78–4.13 (CGGA) (Figure 4I).

Univariable Cox analyses showed that higher risk scores were associated with shorter overall survival in both TCGA (HR = 2.718, 95% CI = 2.372–3.115, *p* < 0.001) and CGGA (HR = 2.060, 95% CI = 1.857–2.285, *p* < 0.001). After adjustment for tumor grade, IDH status, and age, the risk score remained significantly associated with overall survival in TCGA (HR = 1.666, 95% CI = 1.326–2.092, *p* < 0.001) and CGGA (HR = 1.624, 95% CI = 1.430–1.845, *p* < 0.001; Table S3).

### 2.4. Nomogram Establishment

**Figure 5:**
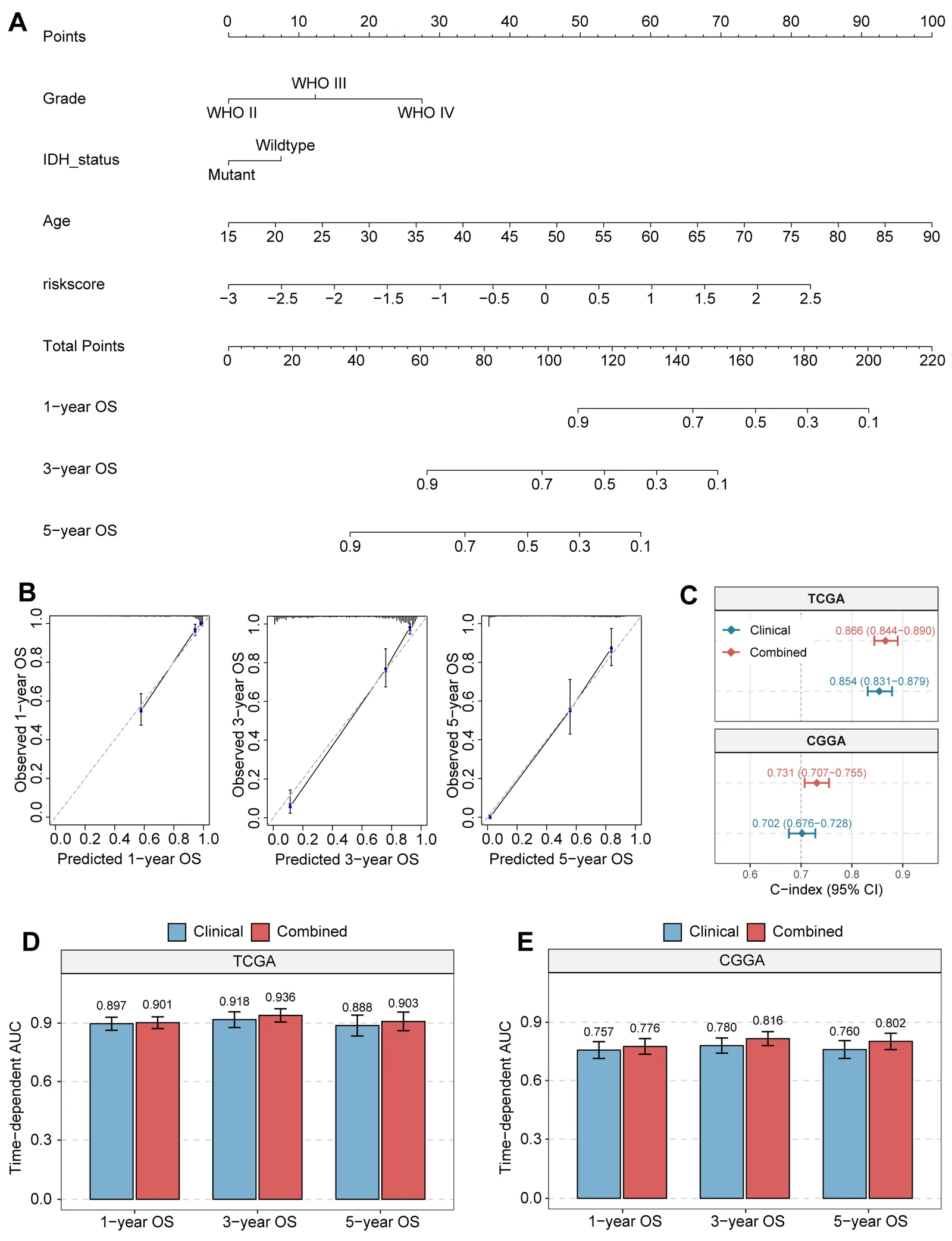
(**A**) Nomogram integrating age, tumor grade, IDH mutation status, and the five-gene risk score to predict 1-, 3-, and 5-year overall survival in the TCGA cohort. (**B**) Bootstrap-corrected calibration curves comparing predicted and observed survival probabilities in TCGA; the diagonal line indicates perfect calibration. (**C**) Comparison of concordance indices (C-indices) between the clinical-only and combined models in TCGA and CGGA, with 95% confidence intervals. (**D**,**E**) Comparison of 1-, 3-, and 5-year time-dependent areas under the receiver operating characteristic curve (AUCs) for the clinical-only model, the five-gene risk-score-only model, and the combined model in TCGA (**D**) and CGGA (**E**).

A nomogram integrating age, tumor grade, IDH mutation status, and the five-gene risk score was developed in TCGA to predict 1-, 3-, and 5-year overall survival (OS; Figure 5A). Bootstrap-corrected calibration showed generally close agreement between predicted and observed survival in TCGA (Figure 5B), whereas external calibration in CGGA showed closer agreement at 5 years than at 1 and 3 years (Figure S3A–C). Adding the five-gene risk score to the clinical model increased the C-index from 0.854 (95% CI, 0.831–0.879) to 0.866 (95% CI, 0.844–0.890) in TCGA and from 0.702 (95% CI, 0.676–0.728) to 0.731 (95% CI, 0.707–0.755) in CGGA (Figure 5C). After correction for optimism, the C-index increased from 0.857 with the clinical model to 0.864 with the combined model in TCGA (ΔC = 0.007). The combined model achieved 1-, 3-, and 5-year AUCs of 0.901, 0.936, and 0.903 in TCGA, compared with 0.897, 0.918, and 0.888 for the clinical model (Figure 5D). After correction for optimism using 1000 bootstrap resamples, the corresponding AUCs were 0.894, 0.919, and 0.887 for the clinical model (optimism 0.001, 0.002, and 0.002) and 0.897, 0.933, and 0.899 for the combined model (optimism 0.003, 0.003, and 0.004). Corresponding AUCs in CGGA were 0.776, 0.816, and 0.802 for the combined model and 0.757, 0.780, and 0.760 for the clinical model (Figure 5E). These findings suggest that the five-gene signature may provide additional prognostic information when integrated with established clinical factors.

### 2.5. Risk Subgroups Demonstrated Distinct Biological Processes

**Figure 6:**
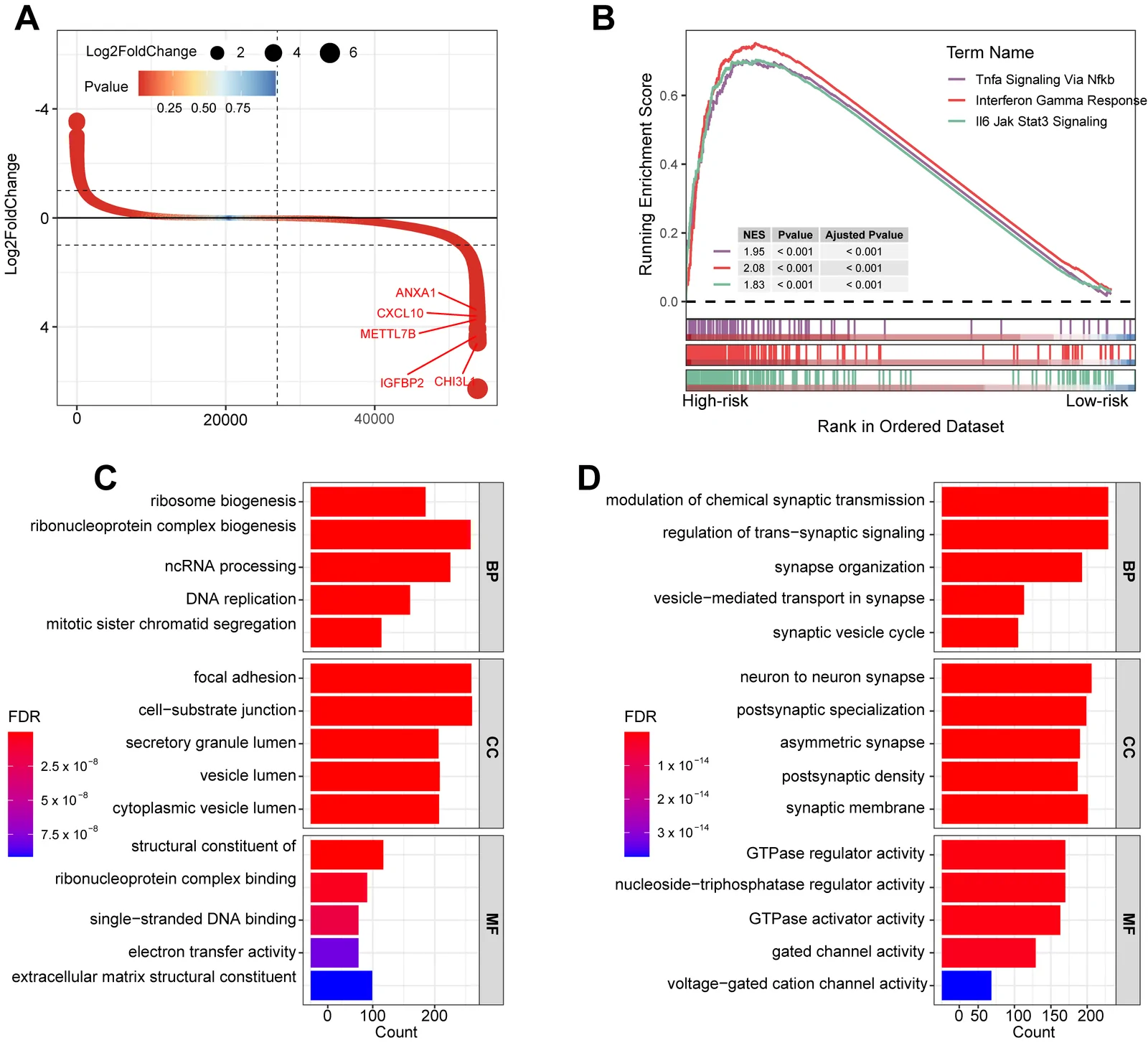
(**A**) Dot plot of differentially expressed genes between the high- and low-risk groups. (**B**) GSEA of representative immune-related gene sets. Genes were ranked by log_2_ fold change (high-risk minus low-risk) in descending order; positive and negative NES indicate enrichment in the high- and low-risk groups, respectively. (**C**,**D**) GO enrichment analyses of genes upregulated in the high-risk (**C**) and low-risk (**D**) groups.

Differential expression analysis identified transcriptional differences between the high and low-risk groups (Figure 6A). GSEA showed enrichment of epithelial–mesenchymal transition, E2F targets, and G2M checkpoint gene sets in the high-risk group (Table S4). Immune-related gene sets, including TNFα signaling via NFκB, interferon gamma response, and IL6/JAK/STAT3 signaling, were also enriched in the high-risk group (all NES > 1 and adjusted *p* < 0.001; Figure 6B and Table S4). GSVA likewise showed higher scores for several immune-related gene sets in high-risk tumors (Table S5), supporting differences in immune-related transcriptional programs. GO analysis of genes upregulated in the high-risk group identified enrichment of terms related to DNA replication and extracellular matrix structural constituent (Figure 6C). Genes upregulated in the low-risk group were enriched for modulation of chemical synaptic transmission, GTPase activator activity, and GTPase regulator activity (Figure 6D).

### 2.6. Correlation of the Risk Score with TME Reprogramming in Glioma

**Figure 7:**
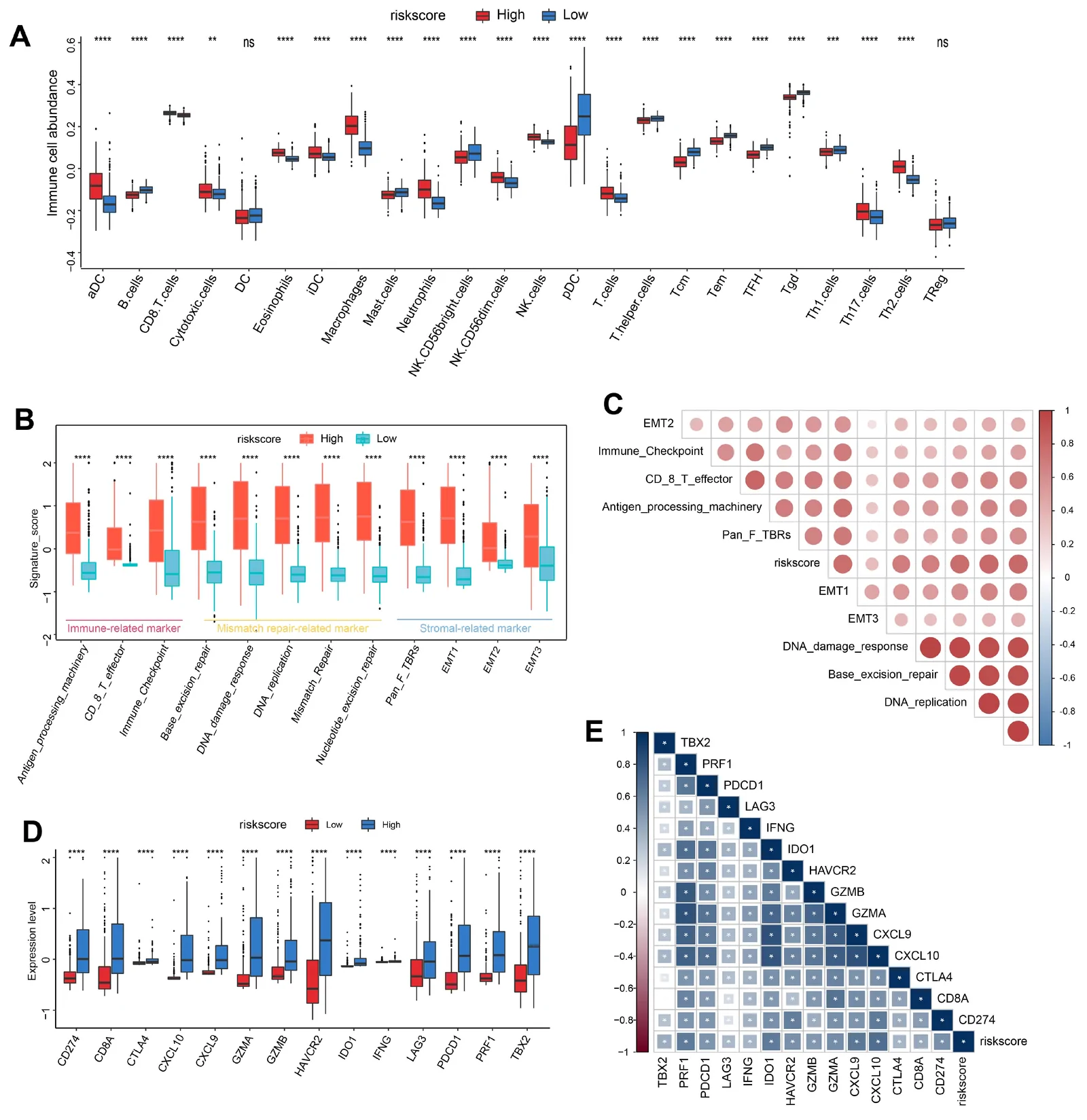
(**A**) Bar plot of immune filtrates calculated by the ssGSEA method and grouped by risk score. (**B**) Bar plot of signature scores calculated by the GSVA method. (**C**) Corplot of the correlation of the risk score and signature score. (**D**) Bar plot of immunotherapy-related marker expression profiles between the high- and low-risk groups. (**E**) Corplot of the correlation of the risk score and immunotherapy-related markers. Data are presented as mean ± SD. Statistical significance was determined by the Wilcoxon rank-sum test. * *p* < 0.05, ** *p* < 0.01, *** *p* < 0.001, **** *p* < 0.0001, and ns indicates no significant difference.

To profile tumor-infiltrating immune cell (TIIC) landscapes, ssGSEA was executed. Macrophage and T-cell abundances were significantly elevated within high-risk glioma samples (Figure 7A). Specifically, the risk signature demonstrated tight positive correlations with both macrophage (R = 0.71, *p* < 0.001) and CD8+ T-cell infiltration layers (R = 0.36, *p* < 0.001; Table S6). Concurrently, gene sets tracking immune activation, stromal elements, and mismatch repair mechanisms were globally upregulated in high-risk patients, showing robust consistency with risk values (Figure 7B,C). Regarding clinical targets for immune checkpoint blockade, key regulatory elements, specifically CD274, HAVCR2, LAG3, CD276, CTLA4, TIGIT, and PDCD1, showed pronounced over-expression in the high-risk cohort (Figure 7D, *p* < 0.0001), further confirmed by Spearman correlation analysis (Figure 7E).

### 2.7. Glioma Somatic Genome Analysis

**Figure 8:**
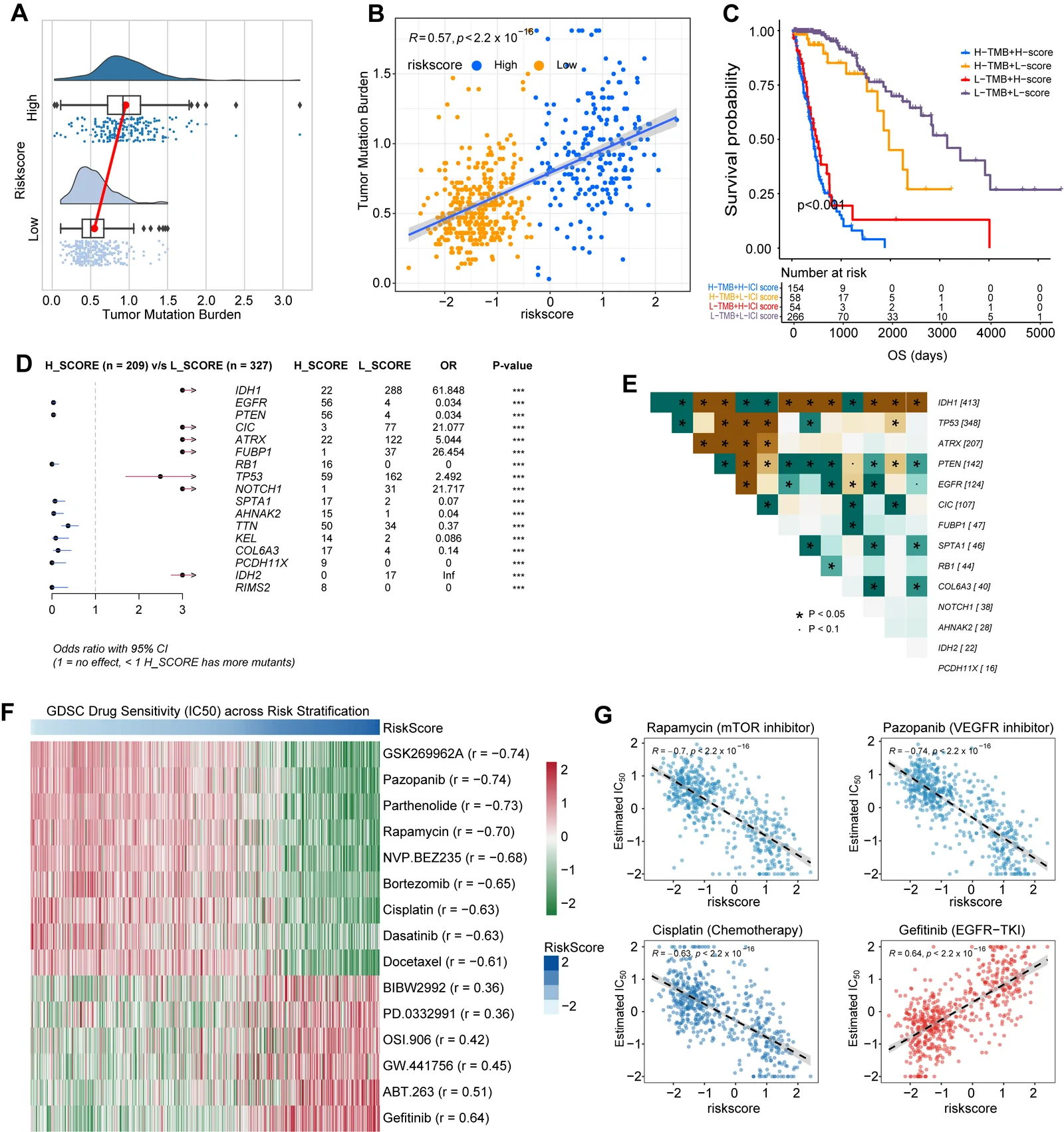
(**A**) Violin plot of TMB distribution between high- and low-risk subgroups. (**B**) Spearman correlation analysis of the risk score and TMB values. (**C**) K-M analysis of glioma prognosis grouped by the risk signature and TMB values. (**D**) Differentially mutated genes (DMGs) between risk subgroups. (**E**) Mutually exclusive or concurrent associations of the DMGs. (**F**) Heatmap showing associations between risk scores and predicted drug sensitivity, with corresponding drug targets. (**G**) Representative scatter plots illustrating the correlations between risk scores and estimated IC50 values for rapamycin (mTOR inhibitor), pazopanib (VEGFR inhibitor), cisplatin (chemotherapeutic agent), and gefitinib (EGFR tyrosine kinase inhibitor). *** *p* < 0.001.

Analysis of tumor mutational burden (TMB) revealed that TMB values positively correlated with the risk signature, showing a significant elevation in the high-risk patient cohort (Figure 8A). The risk score was remarkably correlated with TMB (Figure 8B, Spearman coefficient: R = 0.57, *p* < 2.2 × 10^−16^). Stratification modeling verified the combined prognostic value of the risk score and TMB status in predicting glioma outcomes (Figure 8C). Furthermore, Fisher’s exact test identified 17 genes with distinct mutation frequencies between the high- and low-risk strata (Figure 8D). The somatic interaction network further characterized the exclusivity and co-occurrence patterns among these candidate genes (Figure 8E).

### 2.8. Association Between the Risk Score and Drug Sensitivity

To identify small-molecule perturbations targeting the risk signature, a CMap query nominated butein and imatinib as potential candidates (Table S7). Furthermore, drug sensitivity modeling using GDSC benchmarks was performed to explore the pharmacological profile associated with the risk score (Figure 8F). In silico prediction indicated that high-risk status exhibited strong negative correlations with predicted half-maximal inhibitory concentration (IC50) values for multiple agents, most notably GSK269962A (r = −0.744, FDR < 0.001), pazopanib (r = −0.739, FDR < 0.001), rapamycin (r = −0.697, FDR < 0.001), NVP-BEZ235 (r = −0.680, FDR < 0.001), cisplatin (r = −0.631, FDR < 0.001), and docetaxel (r = −0.612, FDR < 0.001) (Figure 8G), suggesting a potentially higher susceptibility to these agents in high-risk tumors in silico. In contrast, positive associations were predicted for gefitinib (r = 0.645, FDR < 0.001) and afatinib (r = 0.360, FDR < 0.001), implying a relative resistance in the computational models (Figure 8F,G).

### 2.9. Association of SMC3 with the Microenvironment and Cellular States

**Figure 9:**
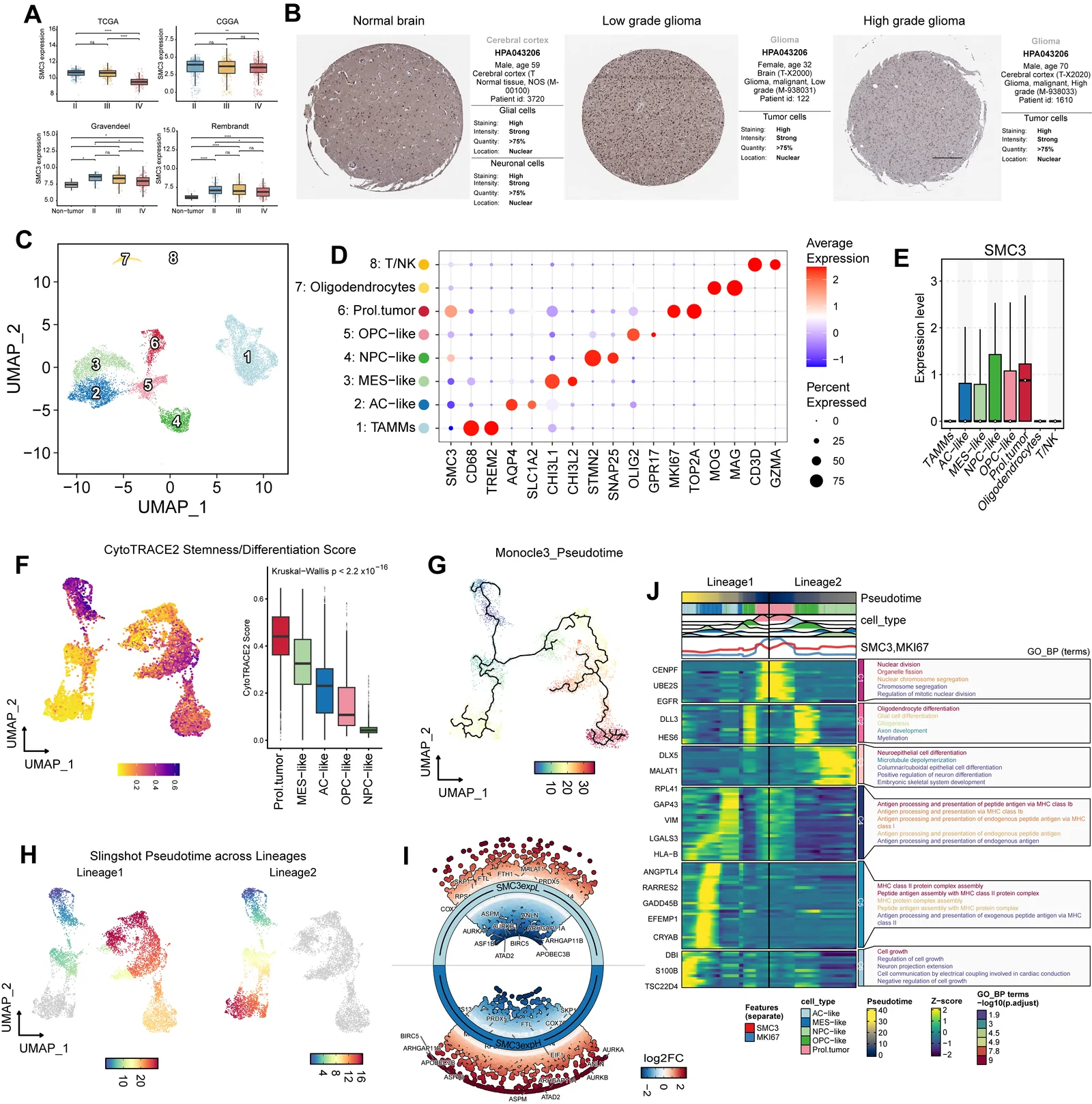
(**A**) SMC3 expression across WHO grades II–IV in TCGA, CGGA, Gravendeel, and Rembrandt. (**B**) Representative immunohistochemical images of SMC3 obtained from the Human Protein Atlas (HPA; antibody HPA043206; patient IDs 3720, 122 and 1610 for non-tumour brain, low-grade glioma and high-grade glioma, respectively). (**C**) UMAP plot of single-cell populations. (**D**) Marker gene expression dot plot. (**E**) SMC3 expression across cell populations. (**F**) CytoTRACE2 score distributions. (**G**) Monocle3 pseudotime trajectory. (**H**) Slingshot pseudotemporal lineages. (**I**) Circular differential expression plot between SMC3-low and SMC3-high malignant cells. (**J**) Lineage-resolved expression and GO biological process dynamics along pseudotime. Pairwise comparisons in (**A**) used two-sided Wilcoxon rank-sum tests with Benjamini–Hochberg correction within each cohort. Differences in (**F**) were assessed using the Kruskal–Wallis test. Significance annotations in (**A**) indicate adjusted *p*-values: * *p* < 0.05, ** *p* < 0.01, **** *p* < 0.0001; ns, not significant.

SMC3 expression was lower in WHO grade IV than in grade II glioma across TCGA, CGGA, Gravendeel and Rembrandt after correction for multiple comparisons. In Gravendeel and Rembrandt, all three tumor grades showed higher expression than non-tumor brain (Figure 9A).

Representative IHC images of non-tumor brain tissue, low-grade glioma, and high-grade glioma were obtained from the HPA database (antibody: HPA043206; patient IDs: 3720, 122, and 1610, respectively). As shown in Figure 9B, all three specimens exhibited strong staining intensity, with more than 75% of the corresponding cell populations showing positive staining. SMC3 staining was predominantly localized to the nucleus across the examined tissue types. UMAP analysis identified distinct malignant transcriptional states—including AC-like, MES-like, NPC-like, OPC-like, and proliferating tumor cells—alongside non-malignant populations (Figure 9C). SMC3 expression exhibited pronounced cell-state-dependent heterogeneity, with the highest expression levels observed in proliferating tumor cells (Figure 9D,E). Furthermore, CytoTRACE2 analysis revealed that proliferating tumor cells possessed the highest developmental potential scores (Figure 9F). Rooted in these high-potency proliferating cells, Monocle3 trajectory inference delineated a branched transcriptional continuum (Figure 9G), which was further corroborated by Slingshot identifying two distinct pseudotemporal lineages (Figure 9H). Moreover, comparative differential expression and marker analyses via the scop framework highlighted the distinct transcriptional hallmarks distinguishing SMC3-low (SMC3expL) and SMC3-high (SMC3expH) malignant subsets, showing robust enrichment of cell cycle- and proliferation-associated drivers in the high-expression group (Figure 9I). Lineage-resolved expression dynamics demonstrated coordinated regulation of SMC3 and MKI67, accompanied by transcriptional programs associated with cell cycle progression, glial differentiation, and immune-related processes along the trajectories (Figure 9J). Together, these findings establish the cell-state-dependent expression of SMC3 and its dynamic modulation across malignant trajectories in IDH-wildtype glioblastoma.

### 2.10. SMC3 Network Perturbation in Malignant Cells

**Figure 10:**
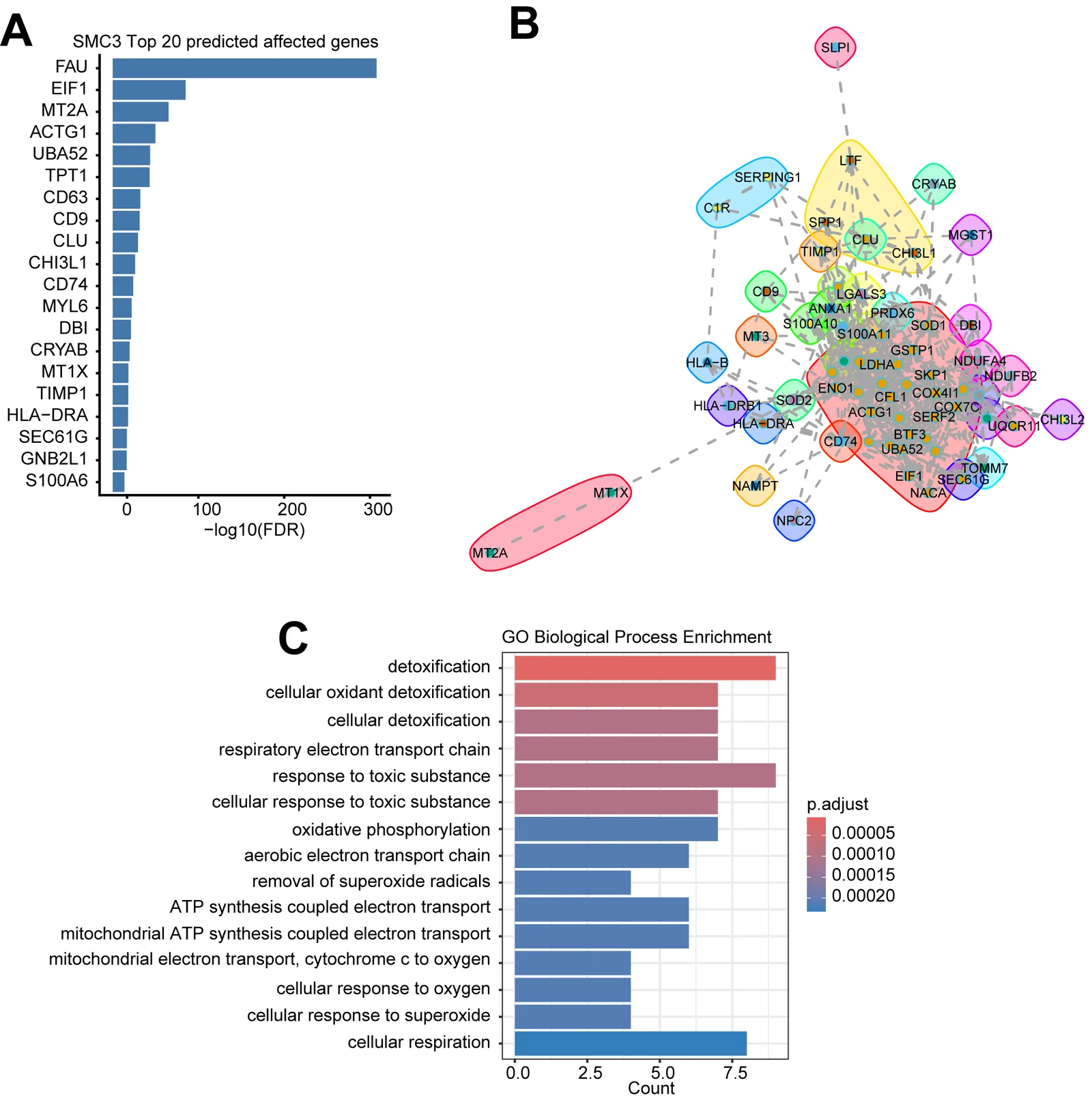
(**A**) Top 20 predicted affected genes ranked by −log_10_(FDR). (**B**) STRING-derived PPI network. (**C**) GO biological process enrichment of all significantly perturbed genes. Bar length indicates gene count; color indicates adjusted *p* value.

To explore the potential regulatory consequences of SMC3 loss, we performed an in silico knockout analysis using scTenifoldKnk in malignant cells from the 10x Genomics subset of GSE131928. The top 20 predicted affected genes, ranked by FDR-adjusted significance, included FAU, EIF1, MT2A, and ACTG1 (Figure 10A). Protein–protein interaction analysis using the STRING database identified interactions among the selected gene products (Figure 10B). GO biological process enrichment analysis was performed on all significantly perturbed genes without separating them by predicted response direction. Enriched terms included cellular detoxification, response to toxic substances, respiratory electron transport chain, and oxidative phosphorylation (Figure 10C).

### 2.11. SMC3 Overexpression Suppresses Glioma Cell Proliferation and Migration

**Figure 11:**
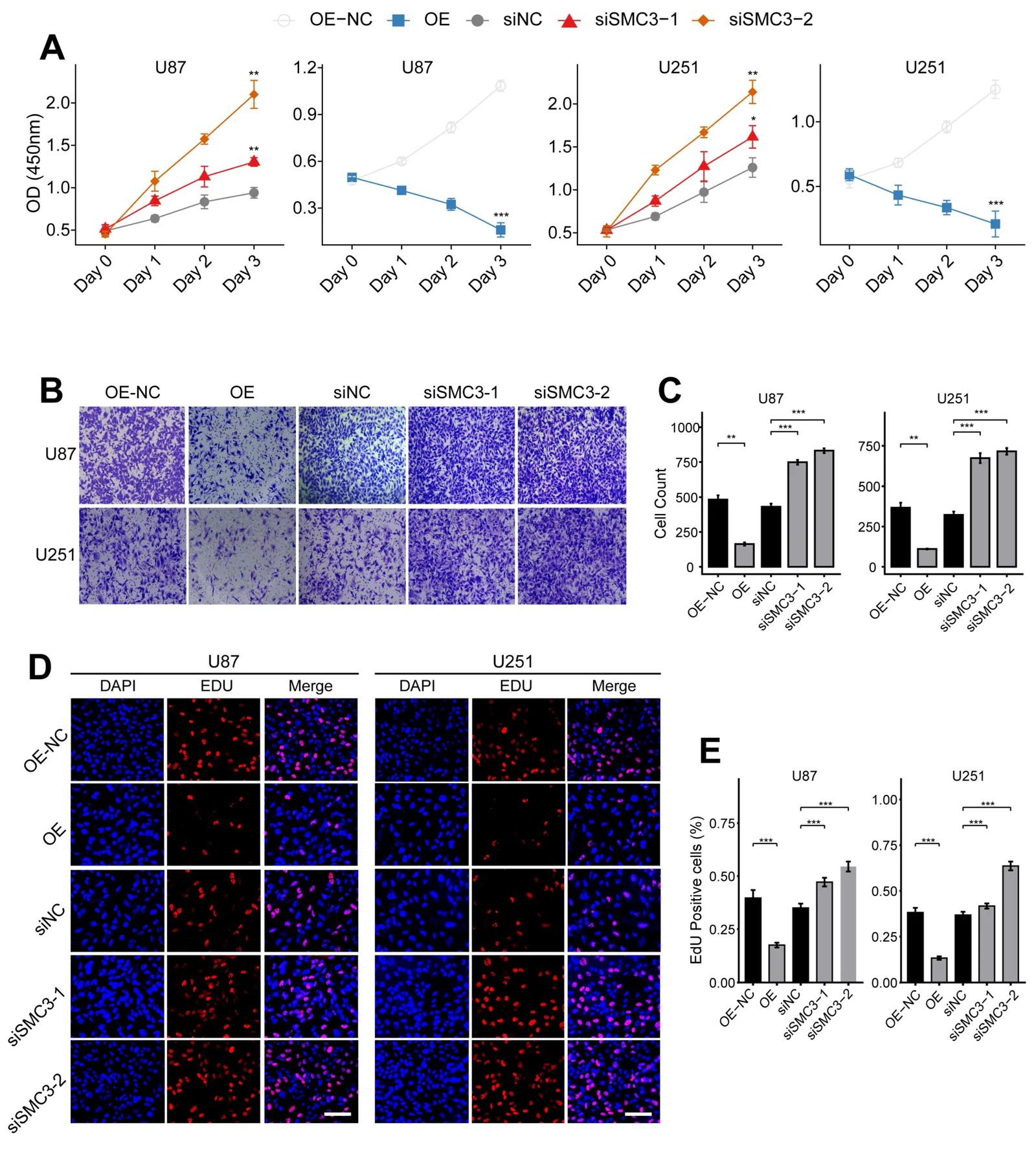
(**A**) Cell viability measured by CCK-8 assays over 3 days in U87 and U251 cells following SMC3 overexpression (OE) or knockdown with siSMC3-1 and siSMC3-2. (**B**,**C**) Representative Transwell migration images (**B**) and quantification of migrated cells (**C**). (**D**,**E**) Representative EdU fluorescence images (**D**) and quantification of EdU-positive cells (**E**). Scale bars = 100 μm. EdU-labelled nuclei are shown in red and DAPI-stained nuclei in blue. Data are presented as mean ± s.d. (*n* = 3). Statistical significance was determined by two-way ANOVA with Dunnett’s test for (**A**); two-tailed Student’s *t*-test for OE versus OE-NC in (**C**,**E**); one-way ANOVA with Dunnett’s test for siRNA groups versus siNC in (**C**,**E**). * *p* < 0.05, ** *p* < 0.01, *** *p* < 0.001.

To investigate the biological functions of SMC3 in glioma cells, we performed gain- and loss-of-function experiments in U87 and U251 cells. The efficiencies of SMC3 overexpression and knockdown were evaluated by qRT-PCR and Western blotting. SMC3 expression was increased in both cell lines by OE-SMC3 compared with the OE-NC control, and was reduced by both siSMC3-1 and siSMC3-2 compared with the siNC control (Figure S4A,B). We then evaluated the effect of SMC3 on cell viability using CCK-8 assays. As shown in Figure 11A, SMC3 overexpression significantly inhibited cell proliferation compared with the OE-NC control group over 0, 1, 2, and 3 days. Conversely, knocking down SMC3 with either siSMC3-1 or siSMC3-2 markedly promoted cell viability. Furthermore, we examined the impact of SMC3 on glioma cell migratory capacity using Transwell migration assays. SMC3 overexpression significantly impaired cell migration, resulting in a distinct reduction in the number of migrated cells compared to the control group (Figure 11B,C). Conversely, SMC3 knockdown by either siRNA clearly enhanced the migratory potential of both U87 and U251 cells (*p* < 0.001). To further assess DNA replication activity, we performed EdU incorporation assays. Aligning with CCK-8 trends, SMC3 overexpression restricted the proportion of EdU-positive proliferating U87 and U251 cells (Figure 11D,E). In contrast, targeted knockdown (siSMC3-1 and siSMC3-2) robustly augmented EdU-incorporating fractions (*p* < 0.001). Together, these experiments showed that SMC3 overexpression reduced proliferation and migration, whereas SMC3 knockdown conferred the opposite effects in U87 and U251 cells.

## 3. Discussion

Recent advancements in DDR-targeted therapies have created new treatment opportunities. Characterizing DDR-related expression patterns may help identify prognostic features and generate hypotheses for therapeutic investigation. The present study analyzed DDR-related DEGs in glioma and developed a prognostic model with great accuracy. Enrichment analyses associated the high-risk phenotype with IFN-γ response and EMT-related transcriptional signatures. Higher risk scores were also associated with macrophage-related transcriptional features, suggesting differences in the immune-related transcriptional environment between risk groups.

Our research aimed to build and validate a 5-gene risk signature (GADD45G, HDAC1, RRM2, SMC3, and WEE1) associated with DDR for predicting glioma patient prognosis and exploring its associations with cancer characteristics and predicted drug sensitivity. HDAC1, RRM2, and WEE1 have been widely reported as critical drivers of glioma progression [23,24,25]. Recent research has also reported an association between elevated SMC3 expression and favorable survival outcomes in glioma, together with potential links to immune-related features [26]. Building on these findings, our study further investigated SMC3 within a DDR-related prognostic framework and explored its expression heterogeneity and functional effects in glioma.

In our analyses, SMC3 expression was lower in GBM compared with grade II and III gliomas, aligning with its potential association with a less aggressive tumor phenotype and favourable prognosis. Single-cell RNA-seq profiling revealed heterogeneous SMC3 expression across malignant cell populations, with prominent enrichment observed in proliferating malignant cells. This pattern aligns with SMC3’s function as a core subunit of the cohesin complex, where it mediates sister chromatid cohesion and chromatin loop extrusion to support high-fidelity DNA replication and maintain genomic stability [27,28]. Pseudotime analysis findings suggest that SMC3 expression may be associated with malignant cell-state heterogeneity. In silico SMC3 knockout identified predicted regulatory perturbations involving genes associated with detoxification and mitochondrial energy metabolism. These findings suggest potential links between SMC3-associated regulatory networks and cellular stress responses [29]. Experimental validation is required to determine whether these processes contribute to the phenotypic effects of SMC3 perturbation.

The prognostic model was developed and validated in two independent cohorts, TCGA and CGGA, and was shown to be highly effective and independent in predicting the survival of patients with gliomas. It has been recognized that a greater AUC value indicates a higher clinical value for the model. The signature established here demonstrated overall AUC values > 0.80 in predicting 3- and 5-year OS of glioma. In comparison to earlier prognostic models for gliomas, the DDR-related signature not only exhibited comparable prognostic accuracy but also facilitated a more precise evaluation of the DDR mechanisms underlying tumor biology, underscoring its reliability and robustness as a prognostic marker [30,31,32,33,34]. In addition, for clinical application, we established nomograms based on CGGA and TCGA glioma data. The AUC value of the nomogram was numerically higher than the AUC value of the DDR-based risk score in predicting patient survival at 1, 3, and 5 years. The results demonstrate the greater advantage of nomogram establishment in predicting patient prognosis in terms of clinical application potential. Consequently, we could conclude from the above that our model had some potential clinical application value and was useful for clinical reasons. In addition, we also identified the independent prognostic value of the risk score that could be incorporated into the nomogram along with the clinical features of the patient for further improvement of the clinical applicability of the nomogram.

This investigation delineates the decisive involvement of DDR-associated expression signatures in modulating glioma pathogenesis and patient survival. It hypothesizes that expression patterns of DDR-related genes could classify glioma samples into distinct molecular subgroups with unique clinicopathological and prognostic profiles. A DDR-related risk model, developed using the LASSO Cox regression, is proposed as a powerful tool to uncover the functional and prognostic significance of DDR in glioma. Our findings demonstrate that higher risk assignment mirrors aggressive neoplastic phenotypes and compromised patient survival. Detailed distribution profiling confirmed a strong positive relationship between increased risk scores and advanced pathological grade. Crucially, reduced risk scores coincided with significant IDH1 and IDH2 mutational enrichment, whereas elevated risk scores corresponded to frequent genomic alterations in EGFR and PTEN. Tumors carrying IDH1 or IDH2 mutations exhibit discrete genetic landscapes that confer favourable survival outcomes relative to non-mutated counterparts [35]. Meanwhile, a great variety of medications, including temozolomide, are effective against IDH-mutated cells due to their low basal NAD+ levels in IDH-mutated cells [36]. The associations between the risk score and predicted drug sensitivity provide an exploratory pharmacological perspective on the identified risk groups. Higher risk scores were associated with lower predicted IC_50_ values for several agents, whereas positive correlations were observed for gefitinib and afatinib. These findings describe model-derived differences and do not demonstrate drug sensitivity or resistance in patients. Gefitinib was evaluated in clinical trials in non-small cell lung cancer (NSCLC), in which mutations in the EGFR intracellular tyrosine kinase domain were revealed in the tumors of patients who showed a significant response to gefitinib treatment [37]. Although gefitinib has been investigated in glioma, its predicted association with the risk score does not establish treatment efficacy or identify patients likely to benefit [38]. Moreover, CMap-based compound nomination and GDSC-based sensitivity modeling address different aspects of drug prioritization, and neither directly measures treatment response in glioma. Their applicability may be limited by differences between the reference experimental systems and human gliomas, including tumor heterogeneity and drug exposure within the central nervous system. Accordingly, these results should be used to prioritize compounds for experimental testing.

Accumulating evidence positions DDR signaling as a critical orchestrator of glioma progression and immune ecosystem remodeling. Under physiological conditions, DDR networks safeguard genome integrity through the precise identification and enzymatic repair of DNA lesions [39]. Dysregulated DDR pathways contribute to enhanced genomic instability, driving tumor aggressiveness and altering the TME [40]. Such dysregulation facilitates immune evasion while reconfiguring the interface between glioma cells and host immune responses, underscoring the translational value of targeting DDR [41]. Here, we characterized DDR-associated risk phenotypes displaying discrete microenvironmental landscapes. In this study, higher DDR-related risk scores were associated with higher transcriptome-derived scores for several immune cell-associated signatures [42].GSEA also identified enrichment of TNFA signaling via NFKB and IFN-γ response gene sets in high-risk tumors. Cytokine-mediated immunity, particularly via IFN-*γ* derived from T lymphocytes and NK cells, represents a primary axis for tuning inflammatory and cytotoxic responses in biological systems [43,44]. Furthermore, GO and KEGG profiling corroborated the hyper-inflamed state of high-risk tumors, showing notable enrichment in complement/coagulation cascades, MHC class II receptor functions, and leukocyte activation programs.

Relevant studies have highlighted that the interplay between DDR mechanisms and macrophages synergistically drives glioma progression. Recent research revealed that the triggering receptor expressed on myeloid cells-2 (TREM2), a transmembrane receptor with potent immunosuppressive functions, plays a crucial role in driving glioblastoma progression. Notably, the HMGB1/TREM2 positive feedback loop intensifies radio-resistance and facilitates immune evasion in glioblastoma by orchestrating the TLR4/Akt signaling pathway [45]. Therefore, we investigated the relationship between DDR and TAMs. By the ssGSEA methods, macrophage and T cell infiltrates were elevated in the high-risk group. In gliomas, a significant proportion of non-tumor cells, accounting for 30% to 50%, resemble tumor-associated macrophages (TAMs) [46]. Meanwhile, the infiltrating macrophages in the TME are typically alternatively activated, specifically, M2 macrophages. These polarized subpopulations of macrophages produce anti-inflammatory molecules, such as TGF-β1 and IL-10, as well as markers that support tissue remodeling and angiogenesis, such as VEGFA, MMP2, and MMP9, which work together and create a matrix that provides the necessary support for the spread and invasion of tumor cells [47,48,49]. By integrating the DDR-related risk signature with microenvironmental profiling, this study identified an association between the risk score and altered immune-related transcriptional features in glioma. These transcriptomic patterns may reflect differences in immune cell composition and functional states, but do not establish effective antitumor immunity or predict therapeutic response. Although combinations of immune checkpoint blockade (ICB) and chemotherapy are being investigated [50,51], the observed associations provide a rationale for further investigation rather than a basis for treatment selection. Their biological significance requires functional characterization, and their predictive value requires validation in cohorts with documented immunotherapy outcomes.

Despite the robust evaluation of the prognosis and immune landscape of the model developed in this study, several limitations must be acknowledged. First, a larger prospective cohort and experimental validation are required to refine the model. Second, our immune analyses were based on computational inference from bulk transcriptomes and therefore could not directly establish immune cell abundance or functional states. The observed associations may be influenced by tumor purity, pathological grade, and IDH status, while the distinction between infiltrating macrophages and brain-resident microglia remains unresolved. Single-cell multi-omics analyses, such as scRNA-seq and spatial transcriptomics, alongside functional immunological assays, will be essential in future studies to resolve cell-type heterogeneity and rigorously test the therapeutic relevance of these DDR-associated immune profiles. Third, although preliminary in vitro experiments provided evidence of phenotypic effects following SMC3 perturbation, the absence of in vivo validation limits conclusions regarding the specificity and physiological relevance of these effects. Fourth, despite conversion of expression values in both TCGA and CGGA to log_2_(TPM + 1), platform- and pipeline-related differences may still affect the stability of the grouping threshold, which requires further testing in larger independent cohorts.

Future studies will employ SMC3 rescue experiment in orthotopic xenograft models and combine multi-omics profiling to elucidate its mechanistic role and therapeutic potential.

## 4. Materials and Methods

### 4.1. Data Acquisition and Processing

Transcriptomic profiles and clinical features were obtained from TCGA and CGGA. Survival analyses of SMC3 and GADD45G were performed in the TCGA (*n* = 663) and CGGA (*n* = 989) cohorts, which contained available overall-survival data. The TCGA (*n* = 543) and CGGA (*n* = 598) cohorts, with the required complete survival and clinicopathological data, were used to develop and validate the prognostic model. RNA-seq expression values were converted from FPKM to TPM and log_2_(TPM + 1)-transformed before risk score calculation. Somatic mutation data were retrieved from TCGA. The Gravendeel (*n* = 275) and Rembrandt (*n* = 313) datasets were obtained from the GlioVis database and used to validate SMC3 expression levels.

### 4.2. Development of the DDR-Related Signature

A gene set comprising 557 DDR-associated targets was retrieved from the MSigDB repository and established literature [52,53,54]. The intersection between these DDR-related candidates and the differentially expressed genes (DEGs) identified via the GEPIA2.0 platform was captured for downstream analysis. Univariate Cox regression modeling was initially implemented to isolate prognostic DDR-related genes. Candidate genes were subjected to LASSO-Cox regression in the TCGA cohort using the glmnet R package. Ten-fold cross-validation was performed using partial likelihood deviance as the evaluation metric. Following LASSO-based selection, GADD45G, HDAC1, RRM2, SMC3, and WEE1 were entered into an unpenalized multivariable Cox proportional hazards model using the survival R package. The resulting coefficients were used to calculate the risk score as follows: Risk score = −0.18013 × EXP(GADD45G) + 0.23596 × EXP(HDAC1) + 0.23374 × EXP(RRM2) − 0.42667 × EXP(SMC3) + 0.36504 × EXP(WEE1), where EXP denoted the log_2_(TPM + 1)-transformed expression value of each gene.

### 4.3. Subgroup K-M Survival Analysis

The TCGA-derived coefficients and fixed cutoff were applied to the CGGA set. Concurrently, chi-square cross-tabulations evaluated the correlation matrix between risk stratification and standard clinicopathological features.

### 4.4. Prognostic Nomogram Construction

Based on the TCGA dataset, a predictive nomogram integrating age, pathological grade, IDH mutation, and the 5-gene risk signature was established using R. Model discrimination was quantified via time-dependent ROC analyses and Harrell’s C-index. Internal validation and calibration were performed using 1000 bootstrap resamples to correct for optimism. Furthermore, external validation was performed by applying the TCGA-derived model to the CGGA cohort to evaluate its generalization capability.

### 4.5. Functional Annotation and Immune Profiling

Functional analysis utilized gene set enrichment analysis (GSEA) and gene set variation analysis (GSVA) referencing the MSigDB hallmark dataset (h.all.v7.5.1.entrez.gmt). Additionally, the immune cell infiltrating profile was comprehensively evaluated utilizing both the ssGSEA algorithm.

### 4.6. Genomic Landscape Characterization

Somatic mutation landscapes between risk cohorts were evaluated using the ‘maftools’ R package [55]. Fisher’s exact test compared gene mutation frequencies across groups, adjusting *p* values via the Benjamini–Hochberg FDR method.

### 4.7. Drug Sensitivity Analysis

Potential therapeutic compounds were identified by querying dysregulated individual genes within the Connectivity Map (CMap) network. Furthermore, we implemented the ‘pRRophetic’ pipeline, integrating pharmacogenomics data from the GDSC database with TCGA glioma expression matrices to simulate the half-maximal inhibitory concentration (IC50) of targeted agents.

### 4.8. Collection and Analysis of scRNA-seq Data

Glioma single-cell transcriptomes GSE131928 (10X) were downloaded from TISCH2 database [56]. Primary quality control, and multi-tier cell-type annotations were initially executed by TISCH2 via the standardized MAESTRO workflow. A total of 10 samples were included in the downstream integration. Batch effects across 10 integrated samples were mitigated using Harmony, followed by dimensional reduction and visualization via UMAP based on the top 2000 highly variable genes and 30 principal components.

### 4.9. Single-Cell Trajectory Inference and Differentiation Potential Analysis

To mitigate technical variation arising from sequencing chemistry, single-cell trajectory analyses were restricted to the 10x Genomics subset of GSE131928. Developmental potential was estimated for individual malignant cells using CytoTRACE2 (v0.3.0), with higher scores indicating greater predicted developmental potential. Proliferating tumor cells, which exhibited the highest CytoTRACE2 scores, were designated as the root population for Monocle3-based trajectory inference. The resulting principal graph was used to assign pseudotemporal positions and characterize transcriptional transitions among malignant cell states. Slingshot was additionally used to infer lineage structures and examine lineage-associated transcriptional dynamics.

### 4.10. Virtual Knock Out of SMC3

To determine how specific gene knockout affects a defined cell population, we analyzed scRNA-seq data from the target cells using the gene × cell expression matrix as input for scTenifoldKnk. The method reconstructs a gene regulatory network (GRN) from the data, then performs in silico deletion of the gene of interest. By aligning the perturbed and original GRNs through manifold alignment, we identified differentially regulated genes indicative of the gene’s function.

### 4.11. Cell Culture

U87MG and U251 human glioma cells were sourced from the American Type Culture Collection (ATCC, Rockville, MD, USA). Authentication was performed via short tandem repeat (STR) profiling, and mycoplasma contamination was excluded. Cultures were grown in DMEM (Gibco, Grand Island, NY, USA) supplemented with 10% fetal bovine serum (FBS) and 1:100 penicillin/streptomycin (Invitrogen, Carlsbad, CA, USA) under 5% CO_2_ at 37 °C.

#### 4.11.1. siRNA Transfection and Overexpression

Following overnight culture in 6-well plates (2 × 10^5^ cells/well) to 50–70% density, cells were transfected via Lipofectamine 3000 (Invitrogen, Carlsbad, CA, USA) per standard protocols. Reagents from GenePharma (Shanghai, China) included SMC3-specific siRNAs (siSMC3-1/2; sequences listed in Table S8), control siRNAs (siNC), pcDNA3.1-SMC3 overexpression constructs, and empty vectors (OE-NC).

#### 4.11.2. Western Blotting (WB)

Total proteins from transfected U87 and U251 cells (harvested 48 h post-transfection) were extracted in RIPA buffer with protease/phosphatase inhibitors (P0013B, Beyotime, Shanghai, China) and measured using a BCA kit (P0010, Beyotime). Equal protein lysates (30 μg) resolved by 10% SDS-PAGE were transferred to PVDF membranes. After 5% milk blocking, membranes were probed at 4 °C overnight with anti-SMC3 (1:1000, Abcam, Cambridge, UK) and anti-GAPDH (1:2000, Proteintech, Wuhan, China) antibodies. Chemiluminescent signals, developed using ECL substrate and captured on a Bio-Rad ChemiDoc system, were quantified using ImageJ (v1.50b) to determine relative SMC3/GAPDH expression levels.

### 4.12. qRT-PCR

Standard TRIzol protocols (Invitrogen, Carlsbad, CA, USA) were used to isolate total RNA, with quality verified on a NanoDrop 2000 instrument. Following cDNA synthesis via PrimeScript RT Kit (TaKaRa Bio, Kusatsu, Shiga, Japan), real-time amplification was monitored on a Bio-Rad CFX96 platform using SYBR Green Premix Ex Taq (TaKaRa). Reaction conditions included a 30 s initiation at 95 °C, followed by 40 two-step cycles (95 °C for 5 s, 60 °C for 30 s). Expression changes in *SMC3* relative to GAPDH were derived via the comparative Ct (2^−ΔΔCt^) approach (primers listed in Table S9).

### 4.13. CCK8 Assay

To evaluate cell proliferation, U87 and U251 cells (3 × 10^3^ cells/well) were cultured in 96-well format in triplicate. Daily assessment (0, 1, 2, 3 days) involved adding 10  μL CCK-8 reagent (KeyGEN, Nanjing, China) per well and incubating at 37 °C for 2 h. Optical density at 450 nm was measured using a microplate detection system (Bio-Rad, Hercules, CA, USA).

### 4.14. Transwell Migration Assay

To evaluate cell motility, serum-starved U87 and U251 cells (5 × 10^4^) were seeded into 8 μm Transwell insert chambers, while complete medium containing 10% FBS was added to the lower compartments as a chemoattractant. Following 24 h of culture at 37 °C, non-migrated cells remaining on the upper membrane surface were gently removed. Migrated cells on the lower membrane surface were fixed with 4% paraformaldehyde (15 min) and stained using 0.1% crystal violet (20 min) for quantification.

### 4.15. EdU Proliferation Assay

After 24 h of initial culture, 50 μM EdU solution was added to cells for a 2 h incubation (37 °C). Cell processing included 4% paraformaldehyde fixation, 0.5% Triton X-100 permeabilization, and click reaction labeling using a KeyGEN kit (KeyGEN, Nanjing, China). DAPI counterstained cell nuclei, and fluorescent signals were visualized under a Nikon fluorescence microscope (Nikon Corporation, Tokyo, Japan).

### 4.16. Immunohistochemical Analysis

Representative SMC3 immunohistochemical images were retrieved from the Human Protein Atlas (HPA) database using antibody HPA043206. The selected specimens included normal cerebral cortex (patient ID: 3720), low-grade glioma (patient ID: 122), and high-grade glioma (patient ID: 1610).

### 4.17. Protein–Protein Interaction (PPI) Network Construction

Genes predicted to be perturbed following in silico SMC3 knockout were submitted to the STRING database (https://string-db.org/), with Homo sapiens specified as the organism [57]. The resulting network mapped proteins as nodes and functional interactions as edges.

### 4.18. Statistical Analysis

Analyses and visualization were conducted in R (v4.1.0) and GraphPad Prism (v9.0). Continuous experimental measurements are summarized as mean ± s.d. Genes were selected by LASSO-penalized Cox regression using glmnet. Overall survival was estimated by the Kaplan–Meier method, with between-group differences assessed by log-rank tests. Univariable and multivariable Cox proportional hazards models yielded hazard ratio (HR) and 95% confidence intervals (CI); multivariable models incorporated age, tumour grade and IDH mutation status. Discrimination was quantified using the concordance index (C-index) and time-dependent receiver operating characteristic AUCs calculated with timeROC. Two groups were compared using Student’s *t*-test or Wilcoxon test. Comparisons of multiple groups with their corresponding control used one-way ANOVA with Dunnett’s post-hoc test. Correlations were evaluated using Spearman’s rank coefficient. Multiple testing was accounted for using the Benjamini–Hochberg procedure. Tests were two-sided, and statistical significance was defined as *p* < 0.05 or a false discovery rate (FDR) < 0.05.

## 5. Conclusions

Accordingly, the five-gene DDR-related signature stratified survival outcomes across glioma cohorts and delineated risk-associated differences in the immune-related transcriptional landscape. Functional experiments demonstrated that SMC3 overexpression attenuated glioma cell proliferation and migration, supporting a role beyond its contribution to the prognostic model. Computational drug sensitivity analyses nominated candidates for experimental validation. These findings support the prognostic relevance of DDR-related expression patterns in glioma, while their therapeutic implications remain to be established.

## Data Availability

The datasets presented in this study can be found in online repositories TCGA (https://portal.gdc.cancer.gov/, accessed on 1 March 2026) and CGGA (http://www.cgga.org.cn/, accessed on 1 March 2026).

## Supplementary Materials

The following supporting information can be downloaded at: https://www.mdpi.com/article/10.3390/ijms27219958/s1.
