# Supplementary material for: Identification of a DNA Damage Response-Related Prognostic Signature and Functional Validation of SMC3 in Glioma
Source: Int J Mol Sci. 2026 Nov 6;27(21):9958. doi: 10.3390/ijms27219958 (PMC13764657; doi:10.3390/ijms27219958)
Supplement: Supplementary file 1 [file ijms-27-09958-s001.zip › Supplementary Material Figures.pdf]

Supplementary Material Figures

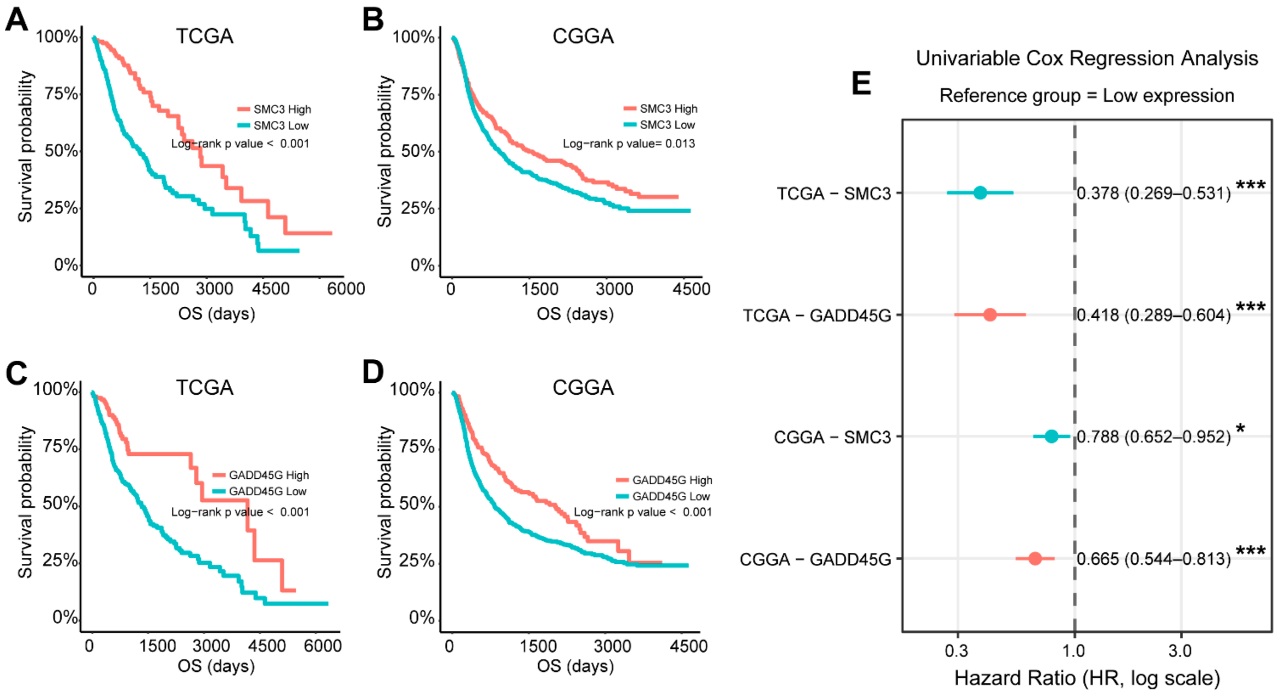

Supplementary Figure S1. (A–D) Kaplan–Meier overall survival (OS) curves for patients stratified by high or low expression of SMC3 in the TCGA (A) and CGGA (B) cohorts, and GADD45G in the TCGA (C) and CGGA (D) cohorts. P values were calculated using the log-rank test. (E) Univariable Cox regression analysis of SMC3 and GADD45G expression in the TCGA and CGGA cohorts. Low expression was used as the reference group. Hazard ratios (HRs) and 95% confidence intervals (CIs) are shown. $*P < 0.05$; $***P < 0.001$.

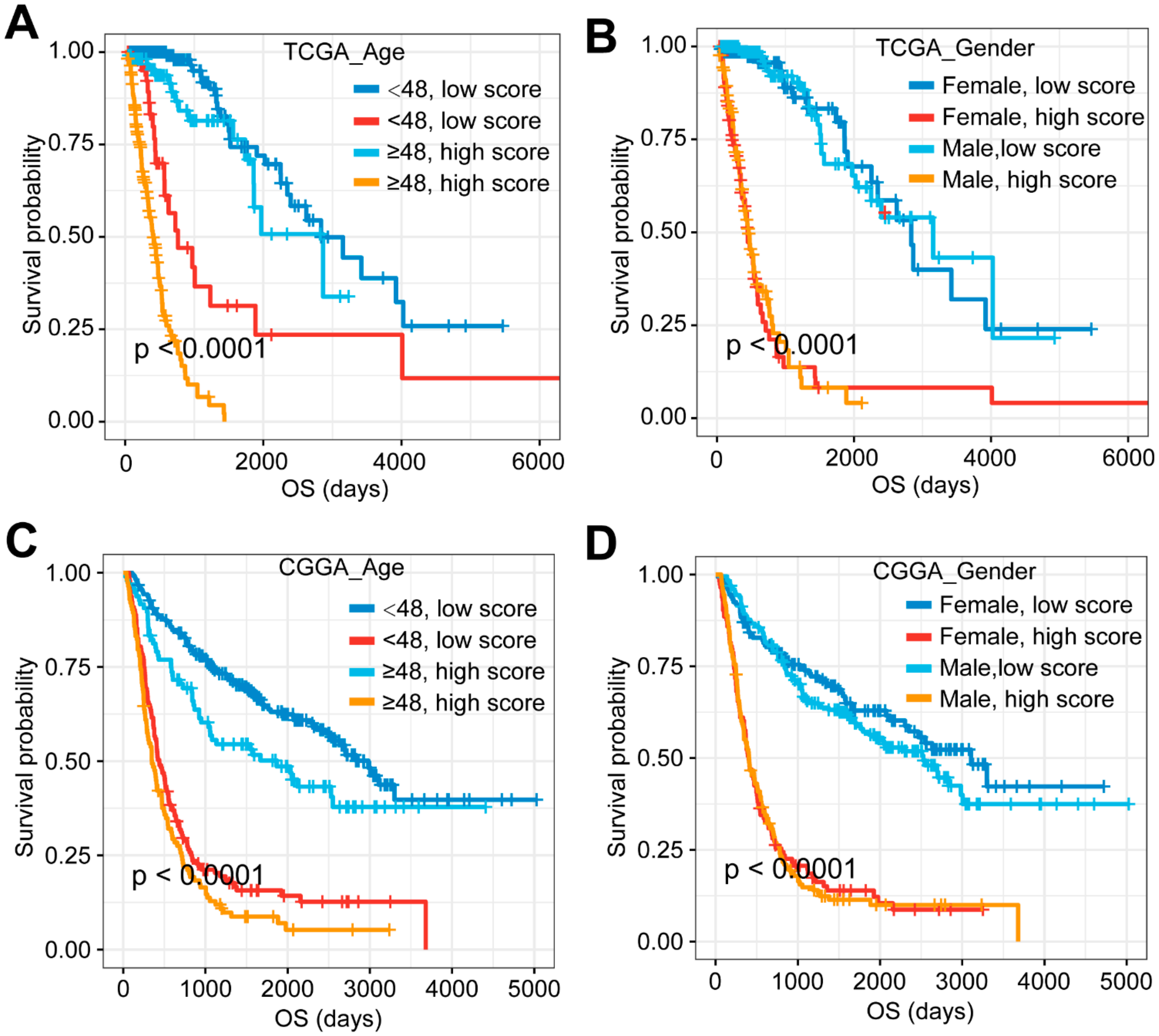

Supplementary Figure S2. (A, C) Overall survival (OS) in the TCGA and CGGA cohorts, respectively, stratified by age (<48 or ≥48 years) and score (low or high). (B, D) OS in the TCGA and CGGA cohorts, respectively, stratified by sex and score. P values were calculated using the log-rank test.

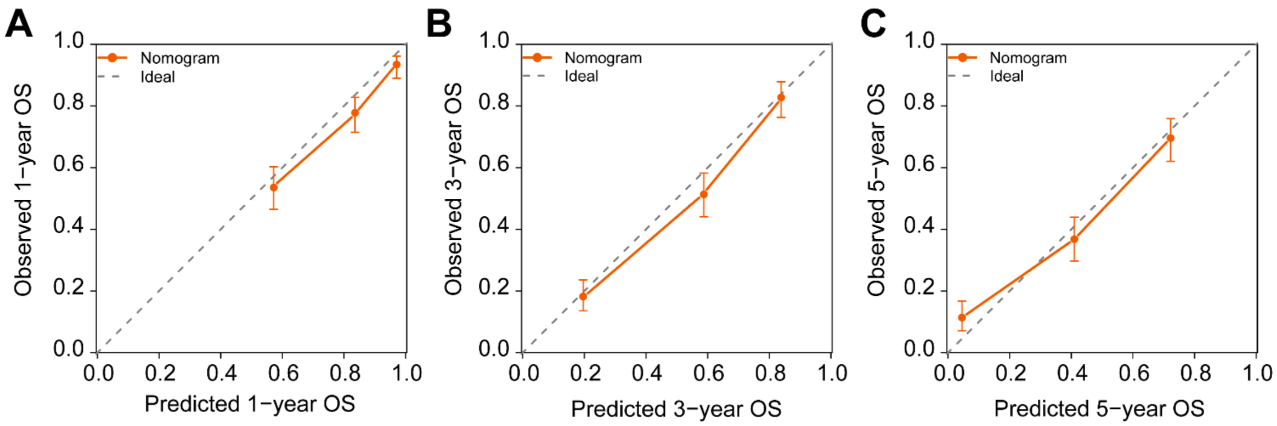

Supplementary Figure S3. Calibration plots compare nomogram-predicted with observed overall survival (OS) probabilities at 1 year (A), 3 years (B), and 5 years (C).

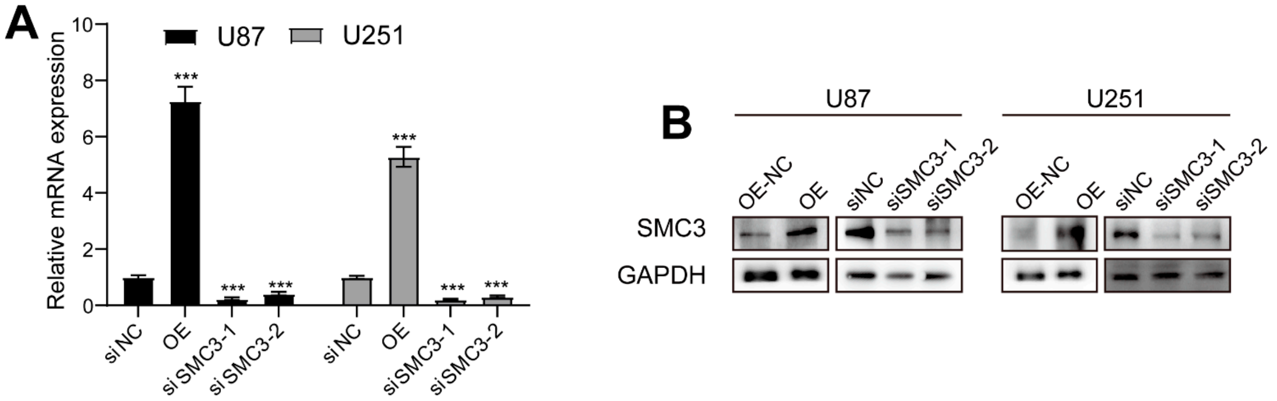

Supplementary Figure S4. (A) qPCR analysis of SMC3 mRNA expression following transfection with the indicated overexpression or knockdown constructs. (B) Representative Western blots showing SMC3 protein levels; GAPDH was used as the loading control. OE, SMC3 overexpression; OE-NC, overexpression negative control; siNC, siRNA negative control; siSMC3-1 and siSMC3-2, SMC3-targeting siRNAs. ***P < 0.001 versus the corresponding control group.
